# Comment on “High doses of biotin can interfere with immunoassays that use biotin-strept(avidin) technologies: Implications for individuals with biotin-responsive inherited metabolic disorders”

**DOI:** 10.1016/j.ymgmr.2019.100506

**Published:** 2019-08-22

**Authors:** Julien Favresse, Damien Gruson, Damien Gheldof

**Affiliations:** a Department of Laboratory Medicine, Cliniques Universitaires St-Luc, Université catholique de Louvain, Brussels, Belgium; b Department of Pharmacy, Namur Research Institute for Life Sciences, Namur Thrombosis and Hemostasis Center, University of Namur, Namur, Belgium

To the Editor,

We read with interest the publication of Barry Wolf published in the latest issue of *Molecular Genetics and Metabolism* [1]. Biotin interference has become a major problem with some laboratory assays and harmful clinical consequences have been reported.

Using a system to adsorb the biotin in serum/plasma (i.e. streptavidin beads [2], VeraPrep Biotin™ [3], BioT-Filter®) is a method of choice to overcome the interference. Other methods used are less practical, because requiring another platform not using the biotin-strept(avidin) immobilization system or to obtain another blood sampling (washout period). Moreover, there is still no consensus defining the correct washout period to be free from biotin.

The author considered the methods to overcome the biotin interference expensive, laborious, only in trial phases, and only performed at specific laboratories [1]. However, utilization of streptavidin beads has been validated by several teams and is easy to perform [2,4]. The technique can be performed in less than one hour, can be performed in every laboratory (agitation and centrifugation), and is not expensive. As a matter of proof, streptavidin beads from manufacturer can be recycled and a minimal impact on control samples has been pointed out [2,4]. The recent VeraPrep Biotin™ test (Veravas) and BioT-Filter® (UNamur) are even able to process the sample in less than 15 min [3]. Furthermore, the technique can also prevent another emerging interference, namely anti-streptavidin antibodies [5,6] with a prevalence that has been evaluated at 0.6% [7].

In conclusion, convenient techniques exist to overcome the biotin interference and could be available in almost all clinical laboratories. It is also important to remind that the correct reporting of such interferences in clinical settings remains the responsibility of the clinical laboratory.

## Declaration of Competing Interest

None.
